# Therapeutic modulation of protein RBM3 for ischemic stroke treatment

**DOI:** 10.3389/fphar.2025.1555115

**Published:** 2025-03-07

**Authors:** Paulo Ávila-Gómez, Alba Vieites-Prado, Clara Correa-Paz, Lucía Del Pozo-Filíu, Nuria Palomar-Alonso, Francisco Campos, Esteban López-Arias

**Affiliations:** ^1^ Department of Pathology and Laboratory Medicine, Weill Cornell Medicine, New York, NY, United States; ^2^ Brain Plasticity Laboratory, Health Research Institute of Santiago de Compostela (IDIS), Santiago de Compostela, Spain; ^3^ Brain Plasticity Laboratory, Centre for research in molecular medicine and chronic diseases (CIMUS), Universidade de Santiago de Compostela, Santiago de Compostela, Spain; ^4^ Neurological Sciences and Cerebrovascular Research Laboratory, Neurology and Cerebrovascular Disease Group, Department of Neurology and Stroke Centre, Neuroscience Area La Paz Institute for Health Research - idiPAZ (La Paz University Hospital- Universidad Autónoma de Madrid), Madrid, Spain; ^5^ Translational Stroke Laboratory Group (TREAT), Clinical Neurosciences Research Laboratory (LINC), Health Research Institute of Santiago de Compostela (IDIS), Santiago de Compostela, Spain

**Keywords:** brain, hypothermia, ischemia, modulation, neuroprotection, RBM3, stroke, therapy

## Abstract

Several preclinical assays and clinical trials have found hypothermia as an efficient protective treatment for stroke. However, systemic hypothermia impairs several physiological functions being difficult to implement in acute critical patients. A deeper understanding of the mechanisms underlying the therapeutic effects of hypothermia could inspire new treatments based on the protective effects of cold. Furthermore, this could contribute to the reduction of the side effects associated with it. One of the metabolic landmarks of hypothermia is the overexpression of a small subset of shock proteins while global protein synthesis is reduced. Among these cold-shock proteins, RBM3 (RNA-binding motif protein 3) seems to play a central protective role. In physiological conditions, which is involved in the regulation of protein synthesis. In several models of cerebral diseases, *in vitro* and *in vivo*, RBM3 exhibited the ability to mitigate apoptosis or increase neural proliferation. In stroke models, RBM3 has shown specially promising effects attenuating neural damage and enhancing cell survival. Future prospects should be directed towards the design of efficient strategies to modulate RBM3 levels. This mini-review aims to summarize the progress made in understanding the role of RBM3 in cerebral tissue protection, while encouraging efforts to address research gaps, particularly in its modulation and clinical application.

## Graphical Abstract

**Figure:**
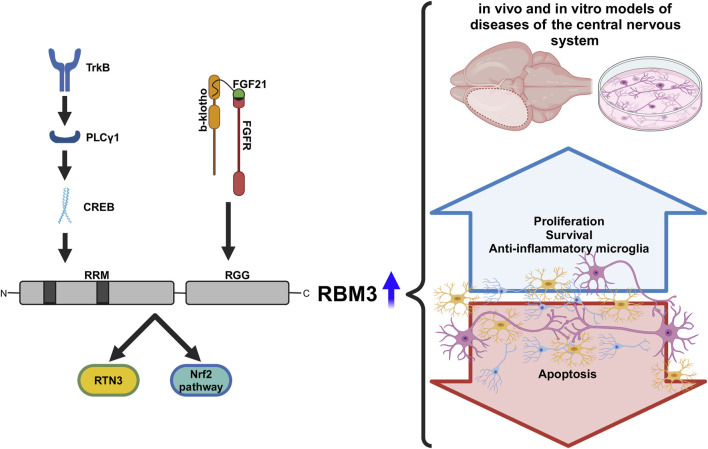
Summary of downstream and upstream pahtway of RBM3, with a detail of its structure. On the right we can see the effects of overexpression of RBM3 in several *in vivo* and *in vitro* models of deseases of central nervous system. Graphical abstract was created with BioRender (biorender.com/)

## 1 Introduction

Ischemic stroke occurs due to insufficient blood flow to cerebral tissue, leading to irreversible damage and, consequently, loss of function and structural support of the affected brain regions. Nowadays, it is still one of the leading causes of death and disability worldwide (World Health Organization, 2024). Approximately 85% of strokes are ischemic (Johnson et al., 2019), and their incidence is expected to increase in the coming years due to the progressive aging of the global population (Béjot et al., 2019).

Despite multiple neuroprotective strategies have been tested (Pérez-Mato et al., 2024), the only available treatment for ischemic stroke in the acute phase is the re-establishment of blood supply, either pharmacologically by intravenous fibrinolytic treatment, or by mechanical thrombectomy. Fibrinolytic treatment consists in rtPA (tissue-type plasminogen activator) injection by infusion that turns plasminogen into plasmin dissolving the clot, and mechanical thrombectomy is a surgical intervention under image guidance with endovascular devices in order to remove the clot (Powers et al., 2019).

Among other clinical parameters, temperature has a deep influence in the outcome of stroke patients (Campos et al., 2012). While hyperthermia worsens the functional outcome (Blanco et al., 2012; Campos et al., 2013; Saini et al., 2009), hypothermia correlates with positive prognosis (Dumitrascu et al., 2016; Miyazawa et al., 2003; Van Der Worp et al., 2007). However, the clinical use of hypothermia carries severe negative side effects such as the reduction of serum K^+^, impairing cardiac function (Mirzoyev et al., 2010); shivering, that requires the use of sedatives and hinders the management of the patient (Nakamura and Morrison, 2011); altered diuresis; disruption of the hemostatic function; immunosuppression; alteration of liver function, or decrease in drug clearance (Polderman, 2009; Wood and Thoresen, 2015). While the clinical use of hypothermia is limited, dissecting its protective mechanisms could inspire the development of new therapeutic strategies.

A hallmark of hypothermic effects over cellular metabolism is a depression of global protein synthesis, while a small subset of cold-shock proteins (CSPs) is overexpressed. Therefore, these CSP could play an important role in its neuroprotective effect. The most studied human CSPs are the cold-inducible RNA-binding protein (CIRP) and RNA-binding motif protein 3 (RBM3). CIRP has been related to increased neuroinflammation in an ischemic stroke model by increasing TNF-α expression and microglial activation resulting in bigger lesion volume (Zhou et al., 2014). Meanwhile, RBM3 has been widely studied and, as discussed in this mini-review, may play a beneficial role in ischemic stroke.

## 2 RBM3 protein

First described by Derry et al. (1995), RBM3 is a member of the glycine-rich RNA-binding protein family with RNA chaperone functions.

### 2.1 Structure, expression and functions

RBM3 contains 157 amino acids (17 kDa) (Derry et al., 1995), and is characterized by a highly conserved RNA recognition motif (RRM) at the N-terminus and a less conserved arginine-glycine-rich domain (RGG) at the C-terminus (Zhu et al., 2016a). The RRM is involved in mRNA processing, while the RGG domain regulates post-transcriptional modifications of RNA and interactions between RNA, DNA, and proteins (Hu et al., 2022).

RBM3 has been identified in embryonic development with a peak in postnatal phase in rats, decreasing after the second week. However, RBM3 can still be detected in the adulthood in the subventricular zone and in the rostral migratory stream, where cell proliferation and migration remain active, as well as in the cerebellum and olfactory bulb where the translation rate is elevated (Pilotte et al., 2009). Despite these facts, RBM3 knockout (KO) mice showed no differences in cerebral or neuronal characteristics compared to wild type that could suggest a redundant mechanism or, as we will discuss below, a prevention to pathophysiological conditions such as hypoxia or hypothermia (Matsuda et al., 2011; Zhu et al., 2019).

Similarly in humans, high levels of RBM3 have been observed in the infant brain (<1 year), especially in the hippocampus and prefrontal cortex; in adult brains, by contrast, RBM3 is nearly absent (Jackson et al., 2018). However, RBM3 has also been detected in higher concentrations in neurons than in glial cells (Jackson et al., 2019), perhaps because the high sensitivity of neurons requires greater prevention of unfavorable conditions.

It has been shown that RBM3 plays a role in several physiological processes at molecular and cellular levels, including: post-transcriptional and post-translational regulation (Sureban et al., 2008), modulation of alternative polyadenylation (Hu et al., 2014), mRNA alternative splicing (Smart et al., 2007), regulation of microRNA (Dresios et al., 2005; Wang et al., 2021), cell stemness and cycle progression (Matsuda et al., 2011), apoptosis (Chip et al., 2011; Zhuang et al., 2017), ER stress (Zhu et al., 2016b), dynamics of synaptic vesicles (Sertel et al., 2021), and circadian cycle (Liu et al., 2013; Sertel et al., 2021).

### 2.2 Relation between RBM3 and temperature

Danno et al. (1997) demonstrated for the first time that mild hypothermia (32°C) for 24 h induced transcription of RBM3 in several immortal human cell lines such as erythroleukemia type K562, hepatocellular carcinoma Hep62, epithelioid cervix carcinoma HeLa, and bladder carcinoma T24. This cold-induced increase has also been found in cortical human neurons differentiated from pluripotent stem cells (Rzechorzek et al., 2015), HEK293 (Zhu et al., 2016b), and human neural lineage cell lines SK-N-SH (Rosenthal et al., 2017), SH-SY5Y (Jackson et al., 2018) and hNSCs (Ma et al., 2024).

In healthy animals, RBM3 protein and mRNA levels were increased in cerebral tissue in rats following four hours of systemic hypothermia at 32°C induced using a rectal-thermostat-controlled electric pad, and remained elevated for a minimum of 6 h post-hypothermia. Focal hypothermia restricted to a single cerebral hemisphere was achieved with a metal coil connected to a cooled water circuit, this device was inserted between skull and temporalis muscle for 24 h, resulting in an increase of RBM3 protein and mRNA levels in the cooled hemisphere compared with the contralateral (normothermic) hemisphere. Therefore, reduction of temperature, whether systemic or localized, can increase RBM3 levels in the brain. Furthermore, similar results were reported in a rodent model of cerebral ischemia (Ávila-Gómez et al., 2022). In addition, pharmacological hypothermia using phenothiazine drugs (chlorpromazine and promethazine, an antipsychotic and a first-generation antihistamine, respectively) also resulted in increased RBM3 expression up to 24 h in a stroke mouse model (Jiang et al., 2022).

In stroke patients, a clinical retrospective study found a negative correlation between basal RBM3 levels in serum and body temperature at admission and at 24 h. Moreover, normothermic (<37.5°C) patients had higher RBM3 values at 24 h than hyperthermic (>37.5°C) patients (Ávila-Gomez et al., 2020). In the same line, analysis of blood samples from patients of the EuroHYP-1 study (a European multicenter, randomized, phase III clinical trial designed to evaluate therapeutic effect of hypothermia in acute ischemic stroke) (van der Worp et al., 2014) did not find significant differences on circulating RBM3 levels in patients who received hypothermic treatment compared with untreated subjects, although rewarming levels tended to be higher in hypothermic patients.

These clinical findings are in agreement with another study in which RBM3 levels increased in the blood of patients with congenital heart disease just after cardiopulmonary bypass (CPB) and therapeutic hypothermia compared with preoperative levels (Rosenthal et al., 2020). In these patients, RBM3 levels were not significantly elevated 24 h after surgery; neither in patients who received CPB but not therapeutic hypothermia. Finally, Wan et al. (2023) detected an elevation in RBM3 blood levels in stroke patients immediately after intra-arterial hypothermia and also 1 day after surgery, an increase that was not identified in the control group without hypothermia treatment.

## 3 RBM3-mediated effect on neuronal development and survival

Despite normal embryonic development in RBM3 KO, the absence of RBM3 causes lack of beneficial effects of cooling: structural and synaptic plasticity in neurons were abolished (Peretti et al., 2021) and neuronal proliferation and differentiation were altered in RBM3 KO mice (Xia et al., 2018; Yan et al., 2019), differentiation of hNSCs into neurons and neuroblasts was diminished after blocking RBM3 with siRNA (Ma et al., 2024), and knockdown of RBM3 by RNAi reduced the number of synapses and impaired novel memory in adult mice (Peretti et al., 2015). As we can see, alterations in RBM3 levels imply changes in the capability of cells and tissues of both neuroprotection and neurorepair processes.

Regarding neuroprotection, several studies have observed a correlation between increased RBM3 levels and an augmented resistance against damage. After staurosporine (STS, a caspase-dependent apoptosis inducer) treatment to induce apoptosis, hypothermia increased RBM3 levels and attenuated caspase-dependent apoptosis in primary murine neurons; blockage of RBM3 by siRNA almost abolished this damage attenuation, while over-expression of RBM3 without hypothermia mimicked the protective effect (Chip et al., 2011; Yang et al., 2017). In a cardiac arrest rat model, hypothermia increased RBM3 levels in certain brain regions resulting in less deficit and less apoptosis in the subgranular zone (SGZ) compared to normothermic controls (Zhang et al., 2021). Similarly, mild hypothermia increased RBM3 levels and decreased apoptosis in SH-SY5Y cells treated with sodium nitroprusside (SNP, a NO donor) with respect to SNP-treated cells under normothermic conditions (Yang et al., 2017).

Hypothermia after hypoxia in SK-N-SH neurons diminished neuronal damage as well as raised RBM3 levels (Rosenthal et al., 2017). According to that, treatment with RBM3 in hypoxic rats improved several neurological functions (Lin et al., 2024).

In different Parkinson disease (PD) models (Yang et al., 2018; Yang et al., 2019), a reduction of apoptosis has also been observed after overexpression of RBM3 in SH-SY5Y cells. Moreover, after adding 1-methyl-4-phenylpyridinium (a dopaminergic neurotoxin) to the medium as PD *in vitro* model, RBM3 overexpression was able to diminish several apoptosis markers (Yang et al., 2018). Yielding consistent results, in PC12 cell lines transfected with fragments of huntingtin with 23 and 74 glutamine repeats as a Huntington’s disease *in vitro* model, RBM3 transfection reduced cell death (Kita et al., 2002).

A reduction in synapse density was observed in wild-type mice immediately after treatment of 45 min at 16°C–18°C (a severe hypothermia), but normal synapse density was restored upon rewarming. This ability to recover was maintained in 5xFAD mice (Alzheimer model) at 2 months of age but lost at 3 months, which mirrors the ability of 5xFAD mice to acutely increase RBM3 in response to hypothermia, but lost at 3 months (Peretti et al., 2015). Similarly, in a prion disease model, synaptic recovery capacity was conserved until 5 weeks post-injection (*w.p.i.*) of prion and lost at 6 *w.p.i.*, similar to the increase of RBM3 by cooling which was annulated at 6 *w.p.i.* (Peretti et al., 2015). These data reinforce the idea that RBM3 is essential in neuronal recovery after adverse conditions. Overexpression of RBM3 by lentivirus-mediated RNAi in this prion model resulted in rescue of synaptic deficit and global translation, improvement of synaptic transmission, reduction of memory deficit, and rise of survival; meanwhile, its blockage reverted the beneficial effects of cooling (Peretti et al., 2015).

Antisense oligonucleotide (ASO) targeting E3a poison exon (an exon within RBM3 pre-mRNA that mediates the degradation of the mRNA) was tested in the intrahippocampal kainate (KA) mouse model of epilepsy resulting in a significant reduction of neuronal loss after status epilepticus induction (Stauffenberg et al., 2024).

The influence of RBM3 in neurorepair was reported too. The same ASO against E3a was also administered in the abovementioned prion model resulting in overexpression of RBM3 and, consequently, in proliferation of pyramidal neurons in CA1 and attenuation of spongiosis (Preussner et al., 2023). Likewise, the overexpression of RBM3 in a hypoxia model in Neural stem cells (NSCs) succeeded in increasing live cells and promoting G1 to S phase transition (Yang et al., 2019).

## 4 RBM3 in ischemic stroke

In ischemic stroke models, *in vitro* and *in vivo*, many examples of activation of neuroprotection and neurorepair can also be found. Experiments with oxygen and glucose deprivation (OGD), as *in vitro* stroke model, revealed activation of neuroprotection when RBM3 was overexpressed, attenuating apoptosis in PC12 cells (Si et al., 2020), in N2a cells (Zhang et al., 2023), and even in organotypic brain slice culture (OBSC); in the last case by stimulating anti-inflammatory microglia proliferation (Zhao et al., 2024). Interestingly, PC12 cells from RBM3 KO presented an increase of apoptosis after OGD respect to cells from WT, moreover, inhibition of RBM3 by siRNA attenuated protective effect of mild hypothermia in N2a cells undergone OGD (Si et al., 2020; Zhang et al., 2023). Analogously, apoptosis induced by OGD in HT22 cells were reverted by adding RBM3 in culture (Lin et al., 2024).

Neurorepair activation has also been reported. For instance, the overexpression of RBM3 without hypothermia in NSPCs stimulated the profileration of these cells after OGD conditions (Zhu et al., 2019).

However, there are some differences in the response to RBM3 depending on the cell source. NSPCs from murine subventricular zone (SVZ) showed increased DCX positive cells when RBM3 was overexpressed with a recombinant form, but no differences in DCX positive cells were observed after OGD between vehicle and RBM3 overexpressed groups. Conversely, NSPCs from subgranular zone (SGZ) presented a rise of neural proliferation and neuronal differentiation when RBM3 was overexpressed in both conditions, but it is noteworthy that this cell type also increased the proliferation and differentiation after OGD in the vehicle group (Zhu et al., 2019).

Both neuroprotection and neurorepair activations were reflected in *in vivo* experiments. Administration of zr17-2 (a small-molecule that targets and modulate RBM3 and CIRP) in ischemic rats enhanced RBM3 protein level, and reduced infarction volume and glial scar formation, enhanced neurogenesis, promoted anti-inflammatory responses, decreased pro-inflammatory markers and improved motor function (Zhao et al., 2024). In line with this, an increase in Rbm3 levels following pharmacological hypothermia was related with better outcomes in stroke mice (Jiang et al., 2022). Reinforcing the importance of RBM3 in cerebral protection under ischemic circumstances, hypoxic-ischemic brain injury in KO mice resulted in an increase of neuronal loss, enhancement of apoptosis in ipsilateral hemisphere and decrease of neural proliferation in the SGZ (Zhu et al., 2019).

Finally, an independent association between RBM3 values at 24 h and good prognosis was found in stroke patients. And more importantly, a variation of RBM3 between admission and 24 h higher than 10% was an independent marker for good prognosis (Ávila-Gomez et al., 2020). Likewise, Wan et al. (2023) also found an independent positive correlation between RBM3 and good outcome at 90 days in stroke patients.

## 5 Non-hypothermic modulation of RBM3

Most of the above-discussed studies accomplished RBM3 overexpression directly by hypothermia or vector transfection, procedures hardly applicable in clinical practice. In search of alternative strategies to induce RBM3 increase, several approaches have been tested, and summarized in Table 1.

**TABLE 1:** Methods to increase RBM3 *in vivo* and *in vitro* (abbreviations: ASO, antisense oligonucleotide; COSC, cortical organotypic slice cultures; FGF21, fibroblast growth factor 21; hNSCs, human neural stem cells; i.v., intravenous; MO, morpholino; OBSC, organotypic brain slice culture; hCNs, human cortical neuronal cells).

Cell line/specie/model	Treatment	Measurement of RBM3	Ref
hCNs	28°C and 32°C	Immunochemistry	Rzechorzek et al. (2015)
HeLa cells	32°C	Western blot	Wellman et al. (2004)
COSC and primary neurons from mice	32°C	Western blot, immunochemistry and RT- PCR	Chip et al. (2011)
HEK293	32°C	Western blot	Zhu et al. (2016b)
HEK293 and SH-SY5Y	32°C	Western blot	Bastide et al. (2017)
Hippocampal slice cultures from mice	32°C	Western blot	Zhu et al. (2016b)
K562 leukemia cells, HepG2, NC65, HeLa and T24	32°C	Northern blot	Danno et al. (1997)
SH-SY5Y	32°C	Western blot	Yang et al. (2017)
SH-SY5Y	32°C	Western blot	Jackson et al. (2018)
SK-N-SH	33.5°C	Western blot and RT-PCR	Rosenthal et al. (2017)
Primary astrocytes from rat	33°C and 36°C	Western blot	Jackson et al. (2015)
Primary cortical neurons from rat	33°C and 36°C	Western blot	
hNSCs	35°C	Western blot and scRNA-seq	Ma et al. (2024)
hNSCs	RBM3 lentivirus	Western blot and qRT-PCR	
OBSC from mice	RBM3 lentivirus	Western blot	Zhao et al. (2024)
C17.2	RBM3 plasmid	Western blot	Yan et al. (2019)
PC12	RBM3 plasmid	Western blot	Si et al. (2020)
SH-SY5Y	RBM3 plasmid	Western blot	Yang et al. (2019)
HT22	RBM3 solution	Western blot	Lin et al. (2024)
HeLa and Hep3B	Hypoxia	Western blot and RT-PCR	Wellman et al. (2004)
NC65	Cycloheximide and puromycin	Northern blot	Danno et al. (1997)
Flp-In T-Rex 293	Doxycycline	Western blot	Zhu et al. (2016b)
Primary astrocytes from rat	FGF21, melatonin and liver X receptor (LXR) agonist T0901317	Western blot	Jackson et al. (2015)
BV2	zr17-2	Western blot	Zhao et al. (2024)
HEK293 and primary hippocampal neurons from mice	MO transfection against the 5’ss of RBM3 E3a	Western blot	Preussner et al. (2023)
HEK293	RBM3 E3a CRISPR/Cas9-mediated genome editing	Western blot and RT-PCR	
C57Bl6/J mice	16°C–18°C	Western blot	Peretti et al. (2021)
C57Bl6/J mice	TrkB agonist 7,8-dihydroxyflavone	Western blot	
C57Bl6/J mice	TrkB agonist 7,8-dihydroxyflavone	Western blot	Bastide et al. (2017)
Swiss mice	Domoic acid	RT-PCR in extracted brains	Ryan et al. (2005)
tg37 ± mice	M2D and M2Db regions ASO	Western blot	Preussner et al. (2023)
Sprague Dawley rats	32°C and i.v. RBM3 solution	Western blot	Lin et al. (2024)
Sprague Dawley rats	32°C–33°C	Western blot and immunohistochemistry	Zhang et al. (2021)
Sprague Dawley rats	32°C (systemic and brain focalized)	Western blot and qPCR	Ávila-Gómez et al. (2022)
Sprague Dawley rats	Phenothiazine drugs	Western blot and RT-PCR	Jiang et al. (2022)
Prion disease mouse model, Alzheimer’s disease mouse model and WT mice	5′-AMP and 16°C–18°C	Western blot	Peretti et al. (2015)
Prion disease mouse model, Alzheimer’s disease mouse model and WT mice	RBM3 lentivirus	Western blot	
Patients with Congenital Heart Disease	Cardiopulmonary bypass under mild hypothermia	ELISA in serum samples	Rosenthal et al. (2020)
Patients with anterior circulation large vessel occlusion under mechanical thrombectomy	4°C normal saline perfusion	Measured in serum samples, method not specified	Wan et al. (2023)

Certain drugs have been shown to upregulate RBM3 expression in rodents and *in vitro*, such as neurotoxin domoic acid, fibroblast growth factor 21 (FGF21), melatonin and zr17-2 (Jackson et al., 2015; Ryan et al., 2005; Zhao et al., 2024). In contrast, metformin and the adenosine monophosphate (AMP) analog AICAR (5-aminoimidazole-4-carboxamide 1-β-D-ribofuranoside, Acadesine, N^1^-(β-D-ribofuranosyl)-5-aminoimidazole-4-carboxamide) have been shown to inhibit its expression *in vivo* and *in vitro*, both of them activators of 5′adenosine monophosphate-activated protein kinase pathway that finally modify alternative splicing (Laustriat et al., 2015).

FGF21, already approved for clinical trials of metabolic disorders (Shao and Jin, 2022), augmented RBM3 levels in young neuron culture (Jackson et al., 2015). In stroke patients, a strong relationship was found between serum FGF21 levels on admission and RBM3 levels 72 h after admission. Moreover, a negative correlation was found between both molecules and maximum temperature, and a positive correlation with good outcome (Ávila-Gómez et al., 2022).

The activation of the TrkB receptor induces upstream RBM3 through PLCγ1 and, in consequence, CREB phosphorylation. In fact, the activation of TrkB with the agonist 7,8-dihydroxyflavone increased RBM3 levels *in vivo* without cooling and induced neuroprotection in prion-infected mice (Peretti et al., 2021).

Downstream, RBM3 binds to reticulon-3 (RTN3) mRNA enhancing its translation, at least in HEK293 cells and in murine hippocampus. Moreover, overexpression of RTN3 promoted neuroprotection in an *in vivo* model of neurodegenerative disease (Bastide et al., 2017) and in *vitro* cell death models (Teng and Tang, 2013); its silencing weakened protective effect of hypothermia or RBM3 overexpression in N2a cells after OGD (Zhang et al., 2023). Conversely, RTN3 has also been identified as neurotoxic since its accumulation provokes the formation of immunoreactive dystrophic neurites, which impairs synaptic plasticity (Hu et al., 2007).

Similarly, Nuclear factor erythroid-derived 2-like 2 (Nrf2) signaling has been found downstream in the RBM3 through growth arrest specific 6. Nrf2 is involved in homeostasis process, mitochondrial function, or anti-inflammation by regulation of antioxidant genes, moreover, its silencing suppressed the anti-inflammatory effects of RBM3 in cell culture (Lin et al., 2024). Likewise, SOX11 pathway has been identified as necessary in neuronal differentiation stimulated by RBM3 (Ma et al., 2024).

A more sophisticated approach involves the use of ASO to promote RBM3 expression by inhibiting the poison exon E3a, as discussed in Section 3 (Preussner et al., 2023; Stauffenberg et al., 2024). This ASO technology is already approved by the Food and Drug Administration (FDA) for Spinal Muscular Atrophy (SMA) (Aartsma-Rus, 2017), making this new approach translational. However, several issues should be considered: both in animal models and in the clinic, the oligo is administered into the cerebrospinal fluid in order to facilitate access to the cerebral tissue. SMA was an orphan and mortal disease, but using this route of administration in stroke or other pathologies could raise ethical concerns. Consequently, alternative approaches have been proposed to vectorize the ASO, avoid its degradation, improve the stability and, ultimately, enable non-invasive administration. This includes approaches such as chemical modifications of the molecule or conjugation with peptides that bind the low-density lipoprotein receptor, aptamers capable to target receptors, or antibodies, among others (Benizri et al., 2019; Ferhat et al., 2024).

## 6 Conclusion

Targeting the RBM3 pathway is a promising strategy to benefit from cooling as treatment for stroke without its negative side effects. However, although multiple neuroprotective strategies have been veotested (summarized in Table 2), several knowledge gaps preclude its use, such as the optimal RBM3 levels required to obtain clinical benefit, the best pharmacological strategy to induce its effect in neuronal tissue, or the optimal therapeutic window.

**TABLE 2:** Effects of RBM3 increasing (abbreviations: ASO, antisense oligonucleotide; HD, Huntington disease; hNSCs, human neural stem cells; i.c.v., intracerebroventricular; i.p., intraperitoneal; MPP+, 1-methyl-4-phenylpyridinium; NSCs, neural stem cells; OBSC, organotypic brain slice culture; OGD, oxygen and glucose deprivation; PC12, rat pheochromocytoma cells; PD, Parkinson disease; ROT, rotenone; SGZ, subgranular zone; SNP, Sodium nitroprusside; STS, staurosporine; SVZ, subventricular zone; tMCAO, transient middle cerebral artery occlusion; i.c., intracerebral).

Specie	Model	Treatment	Findings	Ref
hNSCs		35°C	increased neuronal differentiation	Ma et al. (2024)
hNSCs		RBM3 lentivirus	increased neuronal differentiation	
OBSCs		medium from OGD-stressed BV2 cells treated with zr17-2 before OGD	increase cell viability, decreased cell death and microglia proliferation	Zhao et al. (2024)
COS7 and SK-N-SH	*In vitro* model of HD	RBM3 plasmid	decreased cellular death	Kita et al. (2002)
SH-SY5Y	Apoptosis by SNP	32°C	increased cell viability; decreased apoptosis	Yang et al. (2017)
Primary neurons from mice and PC12	Apoptosis by STS-induction	32°C	decreased apoptosis	Chip et al. (2011)
SK-N-SH	Hypoxia	33,5°C	decreased cytotoxicity and neuronal damage	Rosenthal et al. (2017)
HT-22	OGD	medium from OGD-stressed BV2 cells treated with zr17-2 before OGD	increase cell viability	Zhao et al. (2024)
HT-22	OGD	RBM3 lentivirus	increase cell viability	
OBSC from mice	OGD	RBM3 lentivirus	increased neurogenesis, microglial proliferation and anti-inflammatory response; decreased cellular death	
PC12	Apoptosis by STS-induction	RBM3 pCEP4 vector	decreased apoptosis and necrosis	Chip et al. (2011)
NSCs from mice	Hypoxia	RBM3 plasmid	overcome the cell cycle arrest, decreased cellular death	Yan et al. (2019)
Primary NSCs from mice	Hypoxia	RBM3 plasmid	increased proliferation	
SH-SY5Y	MPP+ *in vitro* model of PD	RBM3 plasmid	decreased apoptosis and damage	Yang et al. (2018)
N2a	OGD	RBM3 plasmid	decreased apoptosis	Zhang et al. (2023)
PC12	OGD	RBM3 plasmid	increased stress reaction; decreased apoptosis and cellular death	Si et al. (2020)
SGZ-NSPCs	OGD	RBM3 plasmid	increased proliferation and neuronal differentiation	Zhu et al. (2019)
SGZ-NSPCs and SVZ-NSPCs	OGD	RBM3 plasmid	decreased apoptosis	
SH-SY5Y	ROT *in vitro* model of PD	RBM3 plasmid	increase cell viability; decreased damage	Yang et al. (2019)
SH-SY5Y	Apoptosis by SNP	RBM3 plasmid	increased cell viability; decreased apoptosis	Yang et al. (2017)
HT22	OGD	RBM3 solution	increased cell viability; decreased inflammatory level, oxidative stress and apoptosis	Lin et al. (2024)
5×FAD mice	Alzheimer’s disease	i.c. RBM3 lentivirus	decreased synapse loss and behavioral deficits	Peretti et al. (2015)
tg37+/− mice	Prion disease	i.c. TrkB agonist 7,8-dihydroxyflavone	decreased behavior deficit and spongiosis	Bastide et al. (2017)
tg37+/− mice	Prion disease	5′-AMP and 16ºC–18°C	increased survival; decreased synapse loss and behavioral deficits	Peretti et al. (2015)
tg37+/− mice	Prion disease	i.c. RBM3 lentivirus	increased survival; decreased synapse loss and behavioral deficits	
tg37+/− mice	Prion disease	i.c. TrkB agonist 7,8-dihydroxyflavone	increased synapsis and memory function; decreased spongiosis	Peretti et al. (2021)
tg37+/− mice	Prion disease	i.c.v. M2D region ASO	decreased neuronal loss and spongiosis	Preussner et al. (2023)
C57BL/6 J mice	Photothrombotic model of ischemic stroke	i.p. zr17-2	increased neurogenesis and anti-inflammatory responses; decreased infarction volume, behavioral deficit, glial scar and pro-inflammatory response	Zhao et al. (2024)
Sprague Dawley rats	Cardiac arrest	32°C–33°C	increased neurogenesis; decreased neurological deficit and apoptosis	Zhang et al. (2021)
Sprague Dawley rats	Cerebral hypoxia	32°C and i.v RBM3 solution	decreased neurological deficit, cellular damage, apoptosis, inflammatory level and oxidative stress	Lin et al. (2024)
Sprague Dawley rats	Stroke model	Phenothiazine drugs	increased functional recovery	Jiang et al. (2022)
Sprague Dawley rats	tMCAO model of ischemic stroke	i.c. RBM3 adeno-associated virus	increased functional recovery	Ávila-Gómez et al. (2022)
